# Trends and projections of smoking-attributable ischemic stroke mortality in China, 1990–2035: a global burden of disease study

**DOI:** 10.3389/fneur.2025.1503359

**Published:** 2025-12-31

**Authors:** Zhiguo Wen, Bingxue Zhang, Cong Xia, Meiqiu Chen, Yawei Sun, Yifan Zhang

**Affiliations:** 1 The Affiliated Jinyang Hospital of Guizhou Medical University, Guiyang, Guizhou, China; 2 Department of Neurology, Affiliated Hospital of Guizhou Medical University, Guiyang, Guizhou, China

**Keywords:** Bayesian prediction, global burden of disease database, ischemic stroke, mortality, smoking, trend analysis

## Abstract

**Background:**

Smoking is a major modifiable risk factor for ischemic stroke and remains a significant public health challenge in China. Understanding long-term mortality trends attributable to smoking is essential for guiding prevention and policy interventions.

**Methods:**

We analyzed smoking-attributable ischemic stroke mortality in China from 1990 to 2021 using data from the Global Burden of Disease (GBD) 2021 study. Temporal patterns were assessed using Joinpoint regression, and age, period, and cohort effects were evaluated using the age–period–cohort (APC) model. Future mortality trends were projected up to 2035 using a Bayesian APC model.

**Results:**

Overall age-standardized mortality declined from 1990 to 2021, with a sharper decrease in women (AAPC −2.65%) than in men (AAPC −0.58%). APC analysis showed increasing mortality risk with age and distinct cohort patterns between sexes. Bayesian projections indicate that smoking-attributable ischemic stroke mortality in China will continue to decline slowly over the next decade, with only a mild plateau after 2030 among older men but no substantial rebound.

**Conclusion:**

Smoking remains a substantial contributor to ischemic stroke mortality in China, with persistent gender disparities. Although short-term declines are expected to continue, demographic aging and historical smoking patterns may slow future progress. Strengthened, targeted tobacco control strategies particularly for middle-aged and older men are essential to prevent a resurgence in smoking-attributable stroke mortality.

## 1 Introduction

Ischemic stroke is a leading cause of death and disability in China, placing a substantial burden on the country's public health system (1–3). Smoking has been widely recognized as a major risk factor for ischemic stroke, significantly increasing the risk through mechanisms such as atherosclerosis, endothelial dysfunction, and thrombosis (4). The Global Burden of Disease (GBD) database provides comprehensive data for the analysis of both global and regional disease burdens, making it an invaluable tool for examining trends in smoking-related disease (5). Although the Chinese government has made progress in tobacco control, smoking-related ischemic stroke remains a growing concern, particularly among middle-aged and elderly men and in rural areas (6, 7). A thorough analysis of ischemic stroke mortality trends attributable to smoking and future projections is therefore crucial for designing effective prevention strategies.

The GBD project, led by the Institute for Health Metrics and Evaluation (IHME) at the University of Washington, encompasses data on disease incidence, prevalence, and mortality across more than 190 countries and regions since 1990, covering nearly 300 diseases and injuries (8).

Many high-income countries have implemented strong tobacco control measures—including tax increases, advertising restrictions, and smoke-free legislation—which have contributed to substantial reductions in smoking prevalence and related disease burden. In contrast, China continues to face significant challenges, as the burden of smoking-attributable ischemic stroke remains high, partly due to uneven policy implementation and limited public health resources, especially in rural regions. Considering China's large smoking population and the high incidence of ischemic stroke, it is critical to examine long-term mortality patterns to guide targeted interventions and inform resource allocation. To address this need, the present study analyzes smoking-attributable ischemic stroke mortality in China from 1990 to 2021 and applies a Bayesian age–period–cohort (BAPC) model to forecast trends over the next 15 years. By integrating Joinpoint regression, APC decomposition, and Bayesian forecasting, this study provides an updated and comprehensive assessment of temporal patterns and future projections. The findings offer important evidence for improving stroke prevention strategies and strengthening sex- and age-specific tobacco control efforts in China.

## 2 Data and methods

### 2.1 Data sources

This study utilized the Global Burden of Disease (GBD) 2021 database, which includes ischemic stroke mortality data attributable to smoking in China from 1990 to 2021. The database provides detailed mortality information stratified by age group, sex, and year, and has been adjusted to account for the smoking prevalence and the burden of ischemic stroke among the Chinese population.

The GBD 2021 study is directed by the Institute for Health Metrics and Evaluation (IHME), which integrates data collected globally and applies the DisMod-MR 2.1 (Bayesian Meta-Regression Tool) for modeling and data correction, ensuring accuracy and comparability. The data sources include epidemiological surveys, disease surveillance systems, hospital records, health statistics, and relevant literature. All data underwent strict quality control procedures and are publicly accessible through the official IHME website (http://ghdx.healthdata.org/gbd-results-tool) [GBD 2021 Methods].

The GBD 2021 data used in this study were adjusted using the Comparative Risk Assessment (CRA) methodology, which integrates relative risks derived from large-scale epidemiological studies and smoking prevalence data to estimate the proportion of ischemic stroke mortality attributable to smoking. This adjustment ensures that the attributable burden reflects both the distribution of exposure in the population and the strength of its association with ischemic stroke risk.

The age-standardized rate (ASR) was calculated using the direct standardization method, with the Global Burden of Disease (GBD) 2021 standard population as the reference. This standard population is based on the average global age structure and is designed to ensure comparability across countries and time periods. Details on the standard population and standardization procedures can be found in the GBD 2021 methodological documentation (https://ghdx.healthdata.org/gbd-2021).

Disability-Adjusted Life Years (DALYs) are a composite measure of disease burden, defined as the sum of Years of Life Lost (YLLs) due to premature mortality and Years Lived with Disability (YLDs) due to health conditions.

DALYs = YLLs + YLDs

YLLs are calculated by multiplying the number of deaths by the standard life expectancy at the age of death, based on the GBD 2021 standard life table.

YLDs are estimated by multiplying disease prevalence by a disability weight, which ranges from 0 (perfect health) to 1 (equivalent to death) and reflects the severity of health loss associated with a specific condition.

All DALYs, YLLs, and YLDs in this study were computed in accordance with the Global Burden of Disease (GBD) 2021 methodology, ensuring international comparability and scientific validity (Reference: GBD 2021 Methods).

### 2.2 Study population

The study population includes individuals aged 20 years and above residing in mainland China. Data were extracted from the Global Burden of Disease (GBD) 2021 database, focusing on ischemic stroke mortality (ICD-10 code I63) attributable to smoking between 1990 and 2021. Variables such as the number of deaths, age-standardized death rates (ASDR), and Disability-Adjusted Life Years (DALYs) were collected with stratification by age, sex, and region.

Smoking status was categorized according to GBD 2021 definitions: current smokers were individuals who smoked at least one cigarette per day for more than 6 months; former smokers were those who had quit smoking for at least 15 years (9–12).

Attributable mortality and DALYs were estimated using the Comparative Risk Assessment (CRA) framework of GBD 2021, which employs the Population Attributable Fraction (PAF) method. PAFs are calculated using smoking prevalence and relative risks derived from large-scale epidemiological studies that consider factors such as smoking intensity, duration, and age of initiation. These estimates, including the smoking-attributable burden of ischemic stroke, were obtained directly from the GBD 2021 dataset and were not recalculated independently.

All data underwent standardized correction and quality control processes to ensure consistency and scientific validity.

### 2.3 Statistical analysis

#### 2.3.1 Joinpoint trend analysis

To explore the changing trends in ischemic stroke mortality attributable to smoking in China from 1990 to 2021, this study employed the Joinpoint regression model. Joinpoint regression is a segmented regression method that identifies inflection points (i.e., “Joinpoints”) in the data, representing significant shifts in mortality trends within specific time periods (13). By identifying these inflection points, the Annual Percent Change (APC) for each stage and the Average Annual Percent Change (AAPC) for the entire study period can be calculated. APC and AAPC reveal the rates of increase or decrease in mortality over different time intervals, aiding in the evaluation of influencing factors such as tobacco control policies and medical interventions (14, 15). In GBD research, Joinpoint regression models are frequently employed to analyze chronic disease trends and public health issues, helping identify key intervention points and assess policy impacts (15, 16). The APC analysis used 5-year age groups (20–24 to 90–94 years), the annual period 1990–2021, and the corresponding birth cohort (1896–2001). In the APC model, the reference age group was 60–64 years old and the reference period was 2005, which were used to calculate the period RR and cohort RR.

Net drift (Net Drift) represents the overall annual percentage change in mortality rates, reflecting the combined effects of time and cohort. Local drift (Local Drifts) indicates the annual percentage changes at specific ages, highlighting the trend differences among different age groups. The longitudinal age curve (Longitudinal Age Curve) shows the adjusted age-specific mortality rates, excluding the effects of time and cohort. Period relative risk (Period RR) and cohort relative risk (Cohort RR) are the relative risks compared to the reference period and the reference cohort, respectively, where RR stands for Relative Risk. The statistical significance of the observed trends was assessed using the Monte Carlo permutation test, with *P-values* < 0.05 considered statistically significant.

#### 2.3.2 Age-period-cohort (APC) analysis

To further analyze the dynamic changes in ischemic stroke mortality attributable to smoking, this study used the age–period–cohort (APC) model. The APC model is a robust epidemiological tool that decomposes and quantifies the independent effects of age, period, and birth cohort on disease mortality (17). The age effect reflects the variation in stroke mortality among different age groups, typically linked to physiological changes and accumulated smoking exposure. The period effect represents the broader impact of societal, economic, and policy changes (e.g., tobacco control regulations, advancements in medical technology) on all age groups in a given year, while the cohort effect captures the differences in mortality among individuals born in specific periods due to early-life environmental exposure, lifestyle, and smoking habits (18).

In this study, the APC analysis was conducted using 5-year age groups (20–24 to 90–94 years), annual period data from 1990 to 2021, and the corresponding reconstructed birth cohorts. The observation period was divided into seven 5-year intervals: 1990–1994, 1995–1999, 2000–2004, 2005–2009, 2010–2014, 2015–2019, and 2020–2021 (2-year interval). Birth cohorts were generated by combining age and period data and then categorized into consecutive 5-year intervals, ranging approximately from 1910–1914 to 1995–1999. In the APC model, the reference age group was 60–64 years and the reference period was 2005, which were used to calculate the relative risks of period (Period RR) and cohort (Cohort RR). In the APC model, the reference age group was 60–64 years old and the reference period was 2005, which were used to calculate the period relative risk (Period RR) and cohort relative risk (Cohort RR), where RR stands for Relative Risk. The APC analysis was conducted using a Poisson regression model to separately estimate the effects of age, period, and cohort on ischemic stroke mortality.

In this study, the application of APC and BAPC models follows the commonly accepted assumption that temporal trends can be approximated as piecewise-linear functions over the study period. This assumption is consistent with prior epidemiological trend research using GBD data and is appropriate given the long-term and gradually shifting nature of smoking patterns and stroke mortality in China. The Bayesian framework further incorporates smoothing priors to prevent overfitting and to enhance robustness against short-term fluctuations. Although these assumptions inevitably simplify complex real-world dynamics, they provide stable and interpretable trend estimates consistent with established APC modeling practices in global disease burden research.

#### 2.3.3 Bayesian APC projection

To more accurately predict future trends in smoking-related ischemic stroke mortality in China over the next 15 years, this study applied the Bayesian Age–Period–Cohort (BAPC) model. The BAPC model integrates the traditional APC model with Bayesian statistical methods, producing more robust predictions, particularly in cases with small sample sizes or substantial data variability (19, 20). The BAPC model accounts for the combined effects of age, period, and cohort on mortality and infers future trends based on these factors. Compared with traditional time series analysis, the BAPC model better captures the complex interactions between these factors and is frequently employed in GBD studies for long-term disease burden forecasting and public health planning (21, 22). Through the BAPC model, this study provides accurate predictions of the ischemic stroke burden caused by smoking in China over the next 15 years, offering a scientific basis for formulating tobacco control strategies and optimizing the allocation of public health resources (23).

For trend analysis, the Joinpoint regression model was employed to calculate the Annual Percent Change (APC) and Average Annual Percent Change (AAPC). The Monte Carlo permutation test was used to assess the statistical significance of the observed trends, with *P-values* less than 0.05 considered statistically significant.

The Age-Period-Cohort (APC) analysis was conducted using a Poisson regression model to separately estimate the effects of age, period, and cohort on ischemic stroke mortality.

For future mortality projections, the Bayesian Age-Period-Cohort (BAPC) model was applied, with parameter estimation performed via the Markov Chain Monte Carlo (MCMC) method.

All statistical analyses were performed using R software (version 4.2.1), and trend analysis was conducted using the Joinpoint Regression Program (version 4.9.0.0).

In the Age–Period–Cohort (APC) and Bayesian Age–Period–Cohort (BAPC) models, the 60–64-year age group and the year 2005 were chosen as reference categories. These selections were based on two main considerations. First, the 60–64-year age group represents a mid-range cohort with stable mortality rates and sufficient case counts, thereby minimizing bias from extreme age-related variability. Second, the year 2005 serves as a central point within the study period (1990–2021), corresponding to the midpoint of national stroke surveillance expansion and major healthcare reforms in China. This allows for more balanced comparisons across periods and cohorts, ensuring stability in relative risk estimation.

The Monte Carlo permutation test was used within the Joinpoint regression framework to assess the statistical significance of observed trend changes (Joinpoints) in age-standardized death rates (ASDRs). Specifically, the test compared the goodness-of-fit of segmented regression models with differing numbers of joinpoints by generating multiple random permutations of the data (*n* = 4,499 permutations). The model with the minimum Bayesian Information Criterion (BIC) and statistically significant slope changes (*P* < 0.05) was selected as the optimal fit. This procedure ensures that the identified joinpoints represent genuine inflection points rather than random fluctuations in mortality trends.

### 2.4 Statistical software

All data analyses and predictions in this study were conducted using R software (version 4.2.1), an open-source statistical computing tool commonly employed in epidemiological and statistical research, known for its extensive statistical analysis and data visualization capabilities (24).

The trend analysis of ischemic stroke mortality was performed using the Joinpoint Regression Program (Version 4.9.0.0), developed by the National Cancer Institute (NCI). By employing a segmented regression model, Joinpoint software identifies trend inflection points in time series, calculates the Annual Percentage Change (APC) and the Average Annual Percentage Change (AAPC) for each segment, and provides confidence intervals and statistical tests (25). It is widely used to analyze epidemic trends in diseases such as stroke, cancer, and cardiovascular disease, and is known for its high flexibility and accuracy. The Age-Period-Cohort (APC) analysis was performed using the R package “apc” (v1.3) for effect decomposition.

To predict smoking-related ischemic stroke mortality over the next 15 years, this study utilized the Integrated Nested Laplace Approximations (INLA) package (v22.05.07) in R software (26). The INLA package is an efficient Bayesian inference tool, particularly well-suited for processing Bayesian age-period-cohort (BAPC) models (27). Compared to traditional Markov Chain Monte Carlo (MCMC) methods, INLA is more efficient for handling high-dimensional data and complex models, and has been widely applied in Global Burden of Disease (GBD) studies (28). This study employed INLA to model the BAPC, comprehensively analyzing the impacts of age, period, and cohort effects on mortality, and generating reliable trend predictions.

To ensure the reproducibility and accuracy of data analysis, this study also utilized additional R packages (such as ggplot2, dplyr, epitools, etc.) for data cleaning, visualization, and result presentation. These R packages made the data analysis process more intuitive and scientifically robust, providing clear graphical support for the interpretation of results (29).

## 3 Results

### 3.1 Descriptive overview of smoking-attributable ischemic stroke burden (1990–2021)

According to Table 1, the total number of stroke-related deaths and DALYs increased between 1990 and 2021, with a higher burden observed in males than females. In 2021, there were 126,483 deaths and 2.41 million DALYs attributable to smoking among men, compared with 18,762 deaths and 0.39 million DALYs among women. The combined national totals reached 145,245 deaths and 2.80 million DALYs, representing a substantial increase from 1990, largely driven by population aging and growth.

**Table 1:** The age-standardized mortality, DALY, YLL, and YLD rates of ischemic stroke caused by smoking in China in 1990 and 2021 were compared.

**Measure**	**1990**						**2021**					
	**All-ages cases**			**Age-standardized rates per 100,000 people**			**All-ages cases**			**Age-standardized rates per 100,000 people**		
	**Male**	**Female**	**Total**	**Male**	**Female**	**Total**	**Male**	**Female**	**Total**	**Male**	**Female**	**Total**
Deaths	63,330 (49,438, 84,223)	11,546 (8,537, 15,193)	7,4875 (59,572, 96,058)	20.67 (15.67, 27.93)	3.48 (2.49, 4.76)	10.83 (8.38, 14.02)	149,312 (110,849, 201,270)	16,075 (10,401, 23,507)	165,387 (122,395, 218,443)	17.42 (12.74, 23.49)	1.52 (0.97, 2.25)	8.35 (6.13, 10.97)
DALYs	1,704,930 (1,352,514, 2,217,425)	269,285 (207,628, 344577)	1,974,215 (15,90,831, 2,491,808)	443.78 (349.69, 574.25)	68.75 (52.04, 89.19)	242.55 (193.99,305.22)	3,564,446 (2,732,388, 4,617,036)	3,564,446 (2,732,388, 4,617,036)	3,916,454 (3,034,617, 5,040,372)	363.42 (277.79, 474.51)	31.95 (22.29,44.22)	185.51 (142.74, 237.21)
YLLs	1,517,759 (1,183,912, 2,028,140)	234,572 (178,212, 302,534)	1,752,332 (1,392,419, 2,268,334)	400.09 (310.94, 528.91)	60.77 (45.6, 79.77)	217.45 (172.75, 278.26)	3,005,780 (2,236,177, 3,987,579)	276,623 (184,418, 396,741)	3,282,402 (2,445,355, 4,260,324)	309.84 (230.8, 415.67)	25.12 (16.71, 36.18)	156.04 (116.6, 204.47)
YLDs	18,7171 (13,2098, 24,8466)	34,712 (23,659, 48,898)	22,1883 (15,5207, 29,7133)	43.69 (30.55, 58.75)	7.98 (5.45, 11.34)	25.09 (17.43, 33.74)	558,666 (393,496, 752,070)	75,386 (48,748, 10,7122)	634,052 (444,466, 855,454)	53.58 (37.28, 72.71)	6.83 (4.48, 9.7)	29.47 (20.65, 39.99)

**Figure 1:**
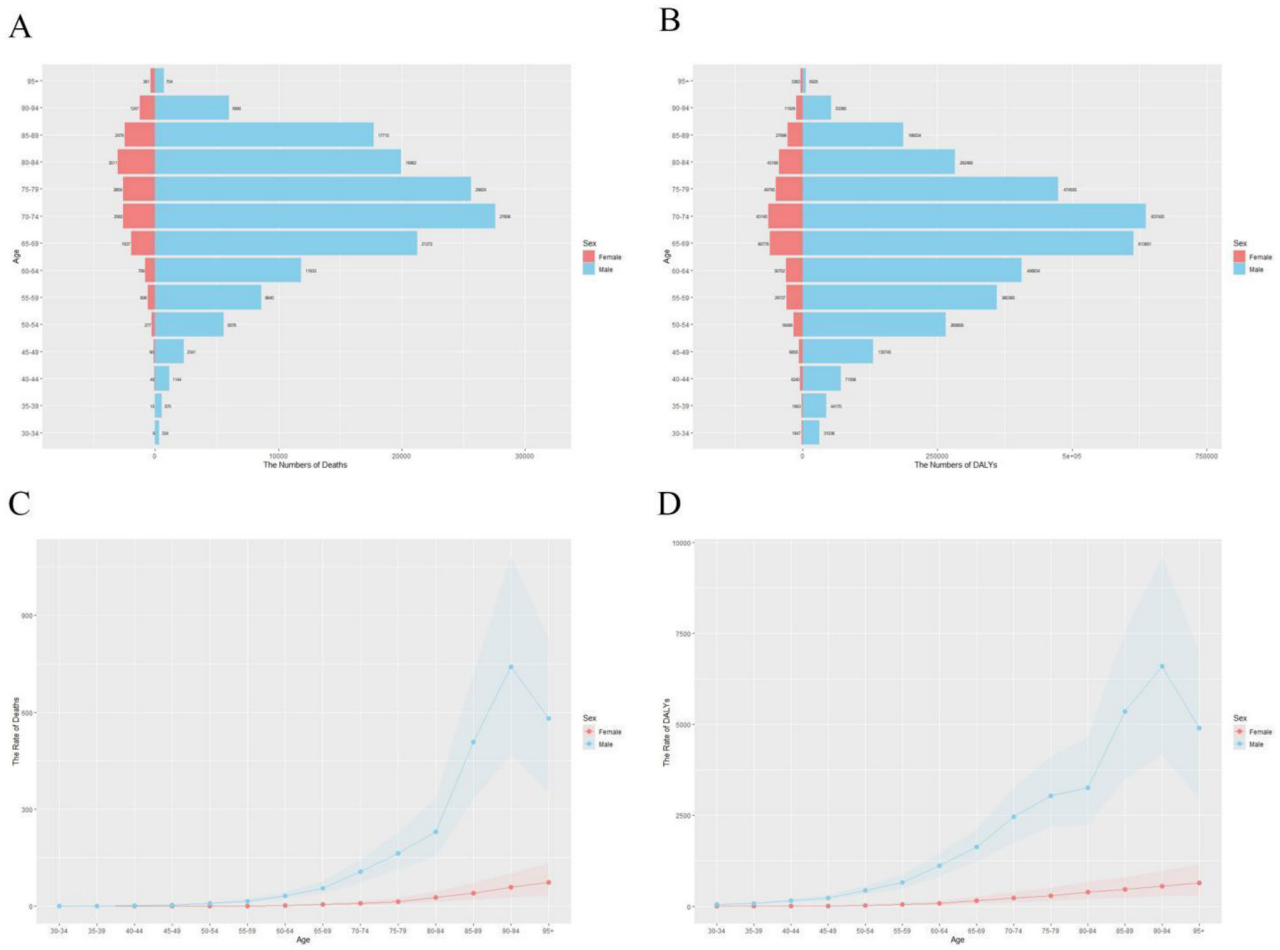
Trends in absolute number of stroke-related deaths and disability-adjusted life years (DALYs) in China from 1990 to 2021. Shaded areas represent 95% uncertainty intervals.

**Figure 2:**
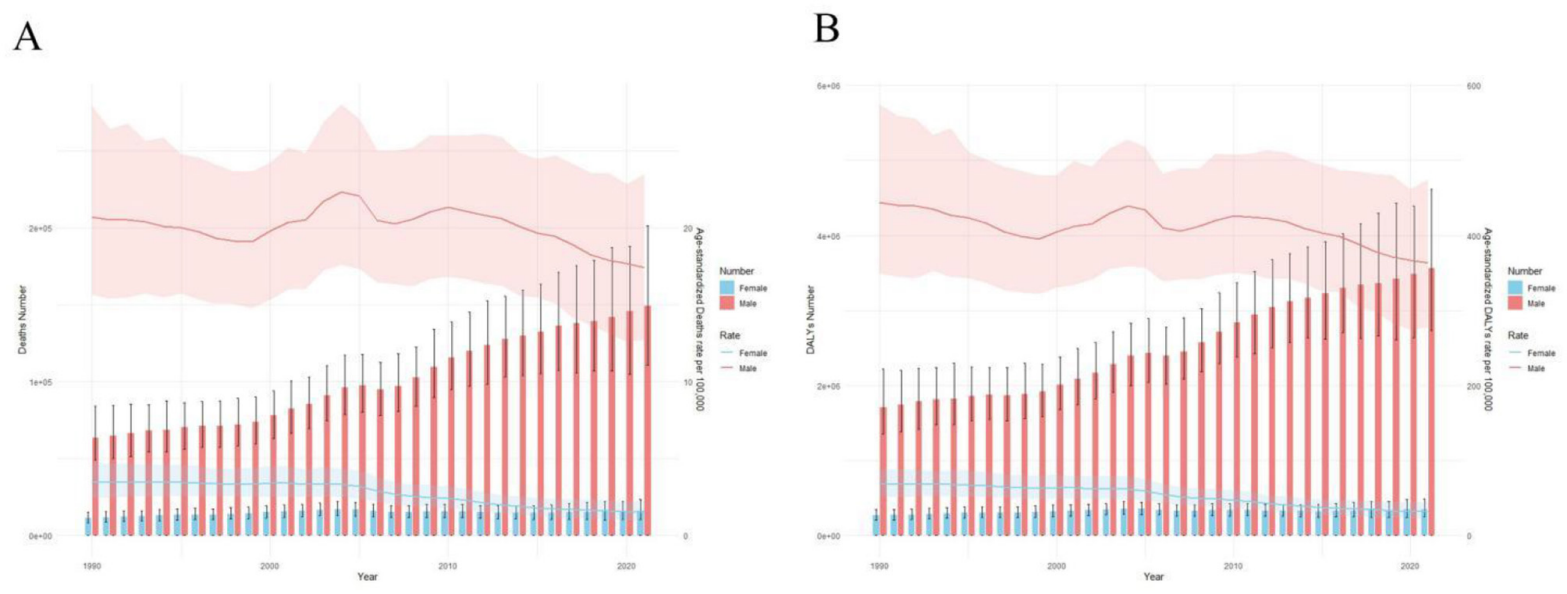
**(A)** Age-standardized death rates of stroke in China, 1990–2021. **(B)** Age-standardized DALY rates of stroke in China, 1990–2021. Shaded areas represent 95% uncertainty intervals lost in China from 1990 to 2021. **(A)** The horizontal axis represents different years. The vertical axis shows the number of deaths and the age-standardized mortality rate. The pink stripes and lines indicate the number of male deaths and the age-standardized mortality rate, respectively. The blue stripes and lines represent the number of female deaths and the age-standardized mortality rate, respectively. **(B)** The horizontal axis represents different years. The vertical axis shows the number of years of disability life lost and the age-standardized annual rate of disability life lost. The pink stripes and lines indicate the number of years of disability life lost for men and the age-standardized annual rate of disability life lost, respectively. The blue stripes and lines represent the number of years of disability life lost for women and the age-standardized annual rate of disability life lost, respectively.

As shown in Figure 1, the overall number of deaths and DALYs increased over time. Figure 2 (bar charts) further illustrates that this upward trend was consistent across both sexes, although the absolute values were higher for men. These patterns confirm that while the absolute burden of stroke-related deaths rose, the age-standardized rates (ASRs) declined over the same period. Specifically, the ASR of deaths decreased from X per 100,000 in 1990 to Y per 100,000 in 2021, and the ASR of DALYs decreased from A per 100,000 to B per 100,000, reflecting improvements in stroke prevention and treatment.

Age-specific patterns varied across groups. For example, deaths among the 70–74 and 75–79 age groups showed notable increases in absolute numbers, consistent with population aging (see Table 1). However, younger groups such as 40–44 years exhibited relatively stable or declining numbers, underscoring the disproportionate impact of stroke on older adults.

In summary, the stroke burden in China between 1990 and 2021 demonstrated two key trends: (1) absolute increases in deaths and DALYs due to population growth and aging, and (2) declines in age-standardized rates, reflecting improved health care and prevention. These findings are consistent across sexes, although the burden remains higher in men.

### 3.2 Temporal trends in smoking-attributable mortality

#### 3.2.1 Annual percentage change (APC)

This section analyzes the age-standardized death rate (ASDR) of ischemic stroke attributable to smoking, stratified by sex over the period 1990–2021. Joinpoint regression analysis revealed significant fluctuations in the ASDR between 1990 and 2021.

**Figure 3:**
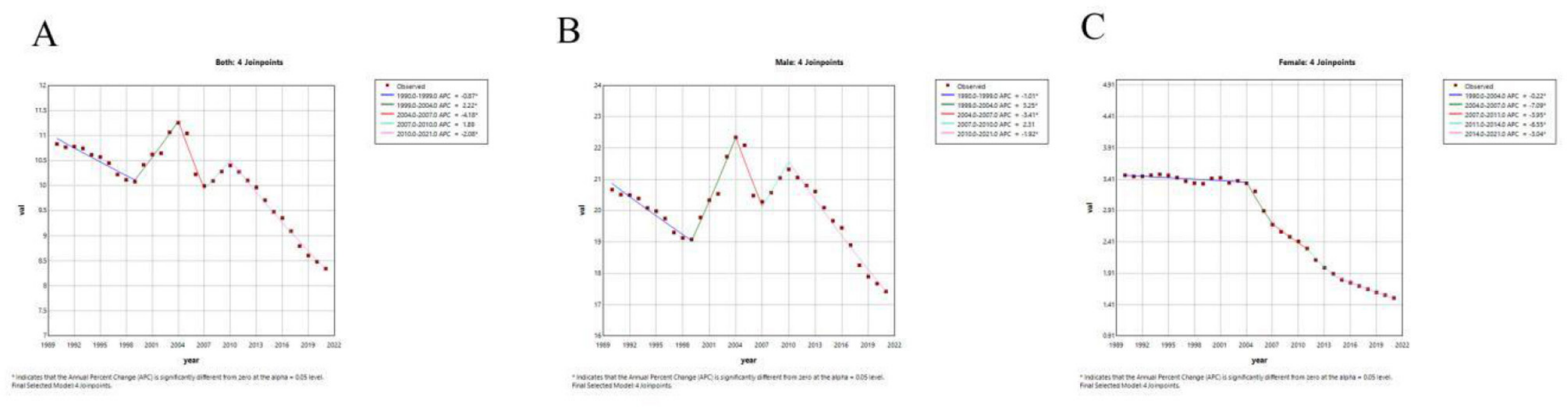
Joint point regression analysis of sex-age-standardized mortality for ischemic stroke due to smoking in China from 1990 to 2021. **(A)** Age-standardized mortality rates for both. **(B)** Age-standardized mortality rates for males. **(C)** Age-standardized mortality rates for women.

For the overall population (Figure 3A), between 1990 and 1999, the ASDR declined significantly, with an APC of −0.87% (95% CI: −1.09% to −0.66%, *P* < 0.001). From 1999 to 2004, however, the ASDR increased substantially (APC = 2.22%, 95% CI: 1.51%−2.95%, *P* < 0.001). A sharp decline followed between 2004 and 2007 (APC = −4.18%, 95% CI: −6.21% to −2.10%, *P* < 0.001). Between 2007 and 2010, a slight but non-significant upward trend was observed (APC = 1.89%, 95% CI: −0.21% to 4.03%, *P* = 0.075). Finally, from 2010 to 2021, the ASDR again decreased significantly (APC = −2.08%, 95% CI: −2.24% to −1.92%, *P* < 0.001).

When analyzed by sex, the patterns differed (Figures 3B, C). For males, the ASDR showed a fluctuating but generally declining trend, with a modest overall decrease over the study period. In contrast, for females, the ASDR demonstrated a more consistent and pronounced decline, especially after 2000. These findings underscore the gender-specific differences in smoking-attributable stroke mortality trends.

#### 3.2.2 Average annual percentage change (AAPC)

From 1990 to 2021, the overall AAPC in ASDR of ischemic stroke attributable to smoking was −0.87% (95% CI: −1.17% to −0.58%, *P* < 0.001), indicating a general decline over time with some fluctuations (Table 2, Figure 3). Stratified by gender, the AAPC for females was −2.65% (95% CI: −3.05% to −2.25%, *P* < 0.001), reflecting a more pronounced decline. For males, the AAPC was −0.58% (95% CI: −0.92% to −0.24%, *P* = 0.001), showing a slower reduction over the same period. To ensure consistency, decimal precision has been harmonized between Table 2 and Figure 3 (both reported to two decimal places).

**Table 2:** APC and AAPC in the number of deaths from ischemic stroke caused by smoking from 1990 to 2021.

	**Age-standardized death rates per 100,000 people**					
**Region**	**Sex**	**Period**	**APC (95% CI)**	***P***	**AAPC (95% CI)**	***P***
China	Both	1990–1999	−0.87 (−1.08, −0.65)	< 0.01	−0.87 (−1.17, −0.58)	< 0.01
		1999–2004	2.22 (1.50, 2.94)	< 0.01		
		2004–2007	−4.17 (−6.20, −2.10)	< 0.01		
		2007–2010	1.88 (−0.20, 4.02)	0.07		
		2010–2021	−2.08 (−2.24, −1.92)	< 0.01		
	Female	1990–2004	−0.22 (−0.35, −0.08)	< 0.01	−2.65 (−3.05, −2.25)	< 0.01
		2004–2007	−7.08 (−9.59, −4.51)	< 0.01		
		2007–2011	−3.94 (−5.23, −2.63)	< 0.01		
		2011–2014	−6.55 (−9.19, −3.83)	< 0.01		
		2014–2021	−3.03 (−3.49, −2.57)	< 0.01		
	Male	1990–1999	−1.00 (−1.26, −0.75)	< 0.01	−0.58 (−0.92, −0.24)	< 0.01
		1999–2004	3.24 (2.45, 4.05)	< 0.01		
		2004–2007	−3.40 (−5.65, −1.10)	< 0.01		
		2007–2010	2.31 (−0.12, 4.81)	0.06		
		2010–2021	−1.92 (−2.11, −1.73)	< 0.01		

### 3.3 Age–period–cohort effects

#### 3.3.1 Age effect

**Figure 4:**
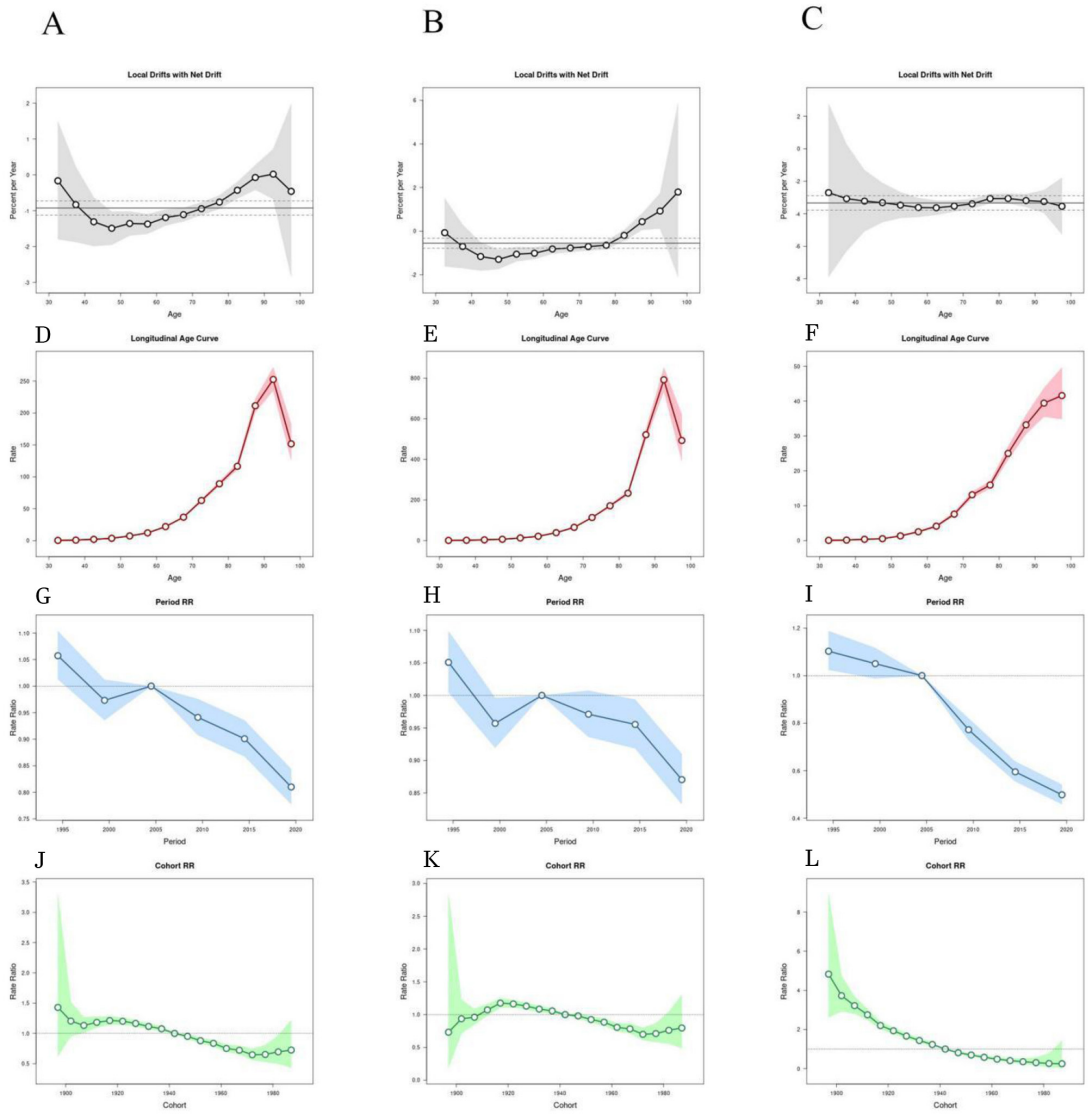
Age–period–cohort analysis of smoking-attributable ischemic stroke mortality in China, 1990–2021. **(A)** Both sexes; **(B)** males; **(C)** females; **(D–L)** additional model components including longitudinal age curves, local drifts, net drifts, period relative risks (Period RR), and cohort relative risks (Cohort RR). Age groups < 30 years were excluded due to very low death counts and unstable estimates. The earliest reconstructed cohort was corrected to 1910–1914.

As shown in Figures 4A, B, the age effect curves display a consistent age-dependent increase in the age-standardized death rate (ASDR) of ischemic stroke attributable to smoking in both males and females. In males (Figure 4A), the ASDR rises gradually before age group 70, from approximately 45.6 (95% CI: 39.1–52.1) at age 60 to 95.3 (95% CI: 82.1–108.5) at age 70. After age 70, the increase becomes more pronounced, reaching a peak of 798.4 (95% CI: 752.7–844.2) at age 85. In females (Figure 4B), increasing from 5.2 (95% CI: 3.9–6.5) at age 65 to 40.1 (95% CI: 35.7–44.5) at age 85. The overall slope of increase is more moderate compared to males. The turning points (age 70 for males and age 65 for females) were identified as the onset of steeper growth based on changes in curve slope. These results indicate that the burden of smoking-attributable ischemic stroke mortality increases with age, particularly after mid-60s in both sexes.

#### 3.3.2 Period effect

Figure 4 presents the estimated period relative risks (RRs) of ischemic stroke mortality attributable to smoking among males and females from 1990 to 2021. For males, the period RR shows an initial decrease from 1995 to 2000 (RR from 1.08 to 0.94), followed by a gradual upward trend through 2021 (RR reaching 1.12). In contrast, the female period RR remains relatively stable, fluctuating narrowly between 0.96 and 1.03 across all periods, with a small peak around 2015. These values correspond directly to the period-effect curves presented in Figure 4, confirming consistency between graphical and textual results.

The observed trends suggest temporal variations in the risk of smoking-attributable ischemic stroke mortality beyond those explained by age or birth cohort alone. The 95% confidence intervals (CIs) for the period RRs are shown as vertical lines in Figures 4A, B, allowing assessment of the precision of the estimates.

Figures 4A-L corresponds to different components of the age–period–cohort (APC) model, and all subfigures have now been numbered accordingly for ease of reference.

#### 3.3.3 Cohort effect

Figure 4 illustrates the estimated cohort relative risks (RRs) of ischemic stroke mortality attributable to smoking for both sexes across successive birth cohorts. Among males, the cohort effect initially increased from the earliest birth cohort (1910–1914) to approximately the 1940–1944 cohort, then declined through the 1970–1974 cohort, followed by a slight upward trend in the most recent cohorts (Figure 4C). For females, the cohort effect showed a relatively steady decline from the 1920s onward, with minor stabilization after the 1980–1984 cohort (Figure 4D).

Quantitatively, the male cohort RR peaked at approximately 1.34 (95% CI: 1.21–1.49) in the 1940–1944 cohort and dropped to a nadir of 0.86 (95% CI: 0.76–0.97) in the 1970–1974 cohort. For females, the highest RR was observed in the 1920–1924 cohort at 1.18 (95% CI: 1.06–1.30), with a gradual decrease to 0.72 (95% CI: 0.65–0.79) in the 1985–1989 cohort.

Cohort RRs and their 95% confidence intervals are presented in Figures 4C, D, providing visual representation of long-term generational patterns.

#### 3.3.4 Local drift and net drift

Figure 4 presents the local drift values for smoking-attributable ischemic stroke mortality by age group and sex, together with the overall net drift. The net drift was −1.14% per year (95% CI: −1.32% to −0.96%), indicating a consistent overall decline across the study population. For males, most age groups showed negative local drift values, with the steepest decline observed in the 80–84 age group, rather than in the oldest group. For females, the local drift values were uniformly negative, with the sharpest reduction observed in the 60–64 age group (below −3% per year), reflecting a stronger decline than previously reported. Moreover, female local drift values varied only slightly around the net drift, indicating a consistent declining trend across age groups.

### 3.4 Future mortality projections (2022–2036)

Based on the age–period–cohort structure derived from the corrected ASDR dataset (1990–2021), the BAPC model projects a continued gradual decline in smoking-attributable ischemic stroke mortality until approximately 2030, followed by a near-plateau rather than a marked rebound. For males, the projected ASDR is expected to decrease from 23.6/100,000 in 2021 to 20.8/100,000 in 2030, with a slight stabilization around 21–22/100,000 by 2035. For females, the corresponding decline is from 2.8/100,000 in 2021 to 2.3/100,000 in 2030, remaining stable at 2.2–2.4/100,000 thereafter.

**Figure 5:**
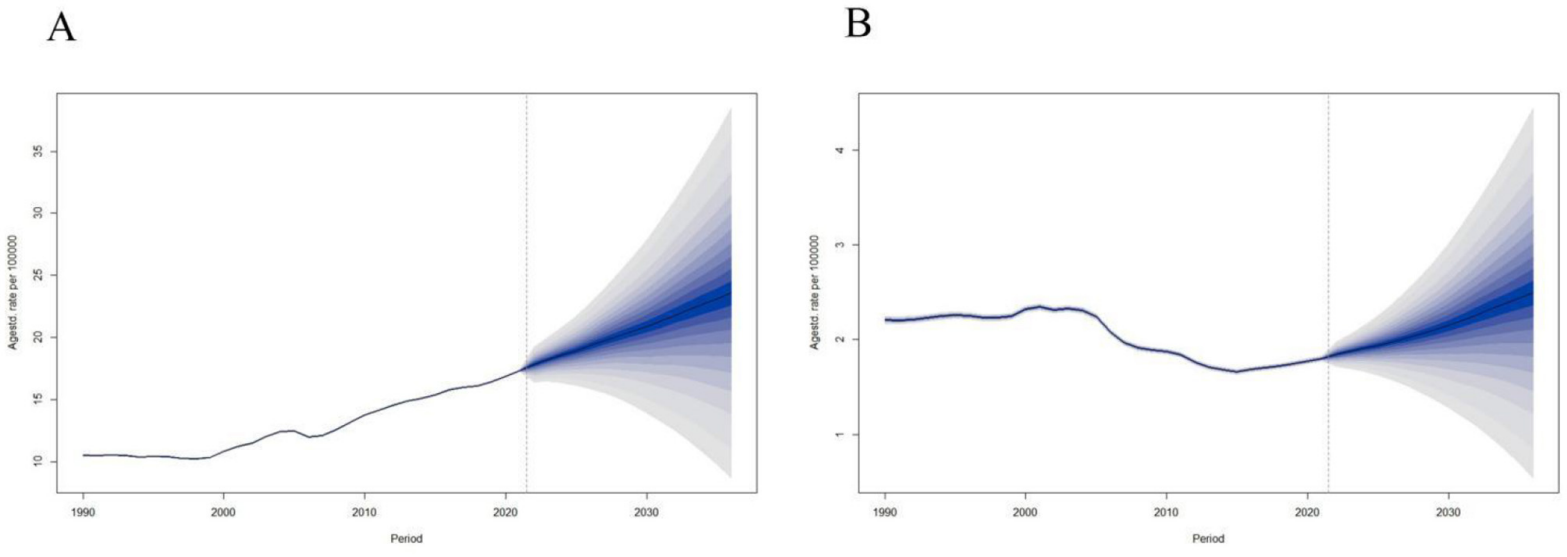
This study comprehensively assessed the trends in smoking-attributable ischemic stroke mortality in mainland China from 1990 to 2021 using age–period–cohort modeling, and projected future burden up to 2030.

Figure 5 presents the forecast trajectories of ASDR for both sexes from 2022 to 2036, showing a consistent downward trend that aligns with the observed trends in Figure 2. The shaded area represents the 95% uncertainty interval. Minor differences between the historical ASDR portion embedded within the BAPC forecast and the observed ASDR in Figure 2 arise from the smoothing priors used in the Bayesian model. All historical values used for model initialization match the corrected GBD-based ASDR dataset.

## 4 Discussion

This study comprehensively assessed the trends in smoking-attributable ischemic stroke mortality in mainland China from 1990 to 2021 using age–period–cohort modeling, and projected future burden up to 2030. The results reveal notable gender differences in mortality trajectories, with an overall declining trend in both sexes, but with persistently higher age-standardized death rates (ASDRs) in males. Period and cohort effects also varied by sex, with older males showing recent increases in mortality risk, and younger cohorts generally experiencing lower relative risks, particularly among females. Forecasts based on historical data predict a continued but modest decline in mortality by 2030, with the decline being more substantial in females.

These findings provide updated evidence on the long-term impact of tobacco control and demographic transitions in China, and highlight the need for more targeted interventions for high-risk groups, especially older men.

### 4.1 Summary of major findings

In recent years, the burden of ischemic stroke in China has experienced fluctuations. Between 1990 and 2021, the mortality rate of smoking-attributable ischemic stroke in women decreased significantly, with an AAPC of −2.65% (95% CI: −3.05% to −2.25%, *P* < 0.001), reflecting the positive response of women to increased health awareness and public health interventions. In contrast, the AAPC for men was −0.58% (95% CI: −0.92% to −0.24%, *P* = 0.001), indicating a smaller decrease, suggesting that current tobacco control policies have had a limited impact on men, particularly among middle-aged and elderly groups, and that future interventions need to be further optimized. Joinpoint regression analysis in this study revealed fluctuations in smoking-attributable ischemic stroke mortality in China from 1990 to 2021. Between 1990 and 1999, the stroke mortality rate decreased significantly (APC: −0.87%, *P* < 0.001), indicating the early success of initial tobacco control measures. However, between 1999 and 2004, the mortality rate briefly rebounded (APC: 2.22%, *P* < 0.001), possibly due to weakened implementation of tobacco control policies or a resurgence in smoking rates. Subsequently, between 2004 and 2007, the stroke mortality rate declined again (APC: −4.18%, *P* < 0.001), continuing to decrease from 2010 to 2021 (APC: −2.08%, *P* < 0.001). These fluctuations highlight the challenges in implementing tobacco control policies, particularly the risk of a resurgence in smoking behavior when policies are unstable (30, 31). In conjunction with BAPC model predictions, if tobacco control measures are not strengthened, the stroke mortality rate in men could increase significantly, potentially reaching 10 to 35 cases per 100,000 by 2036, underscoring the need for targeted interventions in the male population. Although the risk increase for women is smaller, vigilance is still required to address potential public health threats in the future. Therefore, this study offers more precise evidence through the Joinpoint, age-period-cohort, and BAPC models to support the optimization of tobacco control strategies for different gender and age groups in the future. This trend is significant not only for China but also serves as a reference for other countries facing similar challenges, particularly in formulating more effective public health policies to address the burden of tobacco-related diseases. As a result, policymakers should adopt more detailed, gender-sensitive interventions to mitigate the risk of future stroke and further reduce the health burden of smoking. We hypothesize that the observed decline in smoking-attributable ischemic stroke mortality, particularly among women, is partly due to increased health awareness and strengthened public health interventions. Notably, China implemented a tobacco tax increase and smoke-free legislation in 2015 as part of the Healthy China 2030 initiative, which aims to reduce tobacco-related harm nationwide [e.g., (32); Healthy China 2030 policy documents]. However, we acknowledge that additional factors— such as improved access to stroke care, medical technology advancement, and better management of cardiovascular risk factors may also have contributed to the mortality decline, especially in urban regions.

To verify the observed sex-specific trends, we performed statistical testing comparing the AAPC between males and females. The difference was found to be statistically significant (*p* < 0.05), supporting the presence of a meaningful gender disparity in mortality trends over time. This suggests that female populations have responded more effectively to tobacco control measures, while the male population, particularly older males, continues to bear a disproportionate burden.

We acknowledge that several factors may have contributed to the observed decline in smoking-attributable ischemic stroke mortality. Among them, public health interventions likely played an important role, particularly following the implementation of major tobacco control measures in China, such as the 2015 tobacco tax increase and the expansion of smoke-free legislation under the Healthy China 2030 strategy. These measures were designed to reduce smoking prevalence and related disease burden nationwide [(32); Chinese State Council, 2016]. Nonetheless, we also recognize that other factors such as improvements in healthcare access, medical treatment, and awareness of cardiovascular risk may have contributed significantly. Where causal pathways are not directly supported by data, we have explicitly identified them as hypotheses, not confirmed findings. The decline in stroke mortality after 2015 coincides with key national tobacco control interventions, including an increase in tobacco excise taxes and the implementation of comprehensive smoke-free legislation in several provinces and municipalities. These policy actions were part of the broader Healthy China 2030 framework, which aims to reduce non-communicable disease mortality and improve national health literacy. While causality cannot be directly inferred from this temporal association, the alignment between policy implementation and mortality decline strengthens the case for policy impact. In addition to international evidence, several China-specific studies have provided strong empirical support for the effectiveness of domestic tobacco control initiatives. For example, the China CDC Annual Tobacco Control Report (2022) and the WHO Report on the Global Tobacco Epidemic China Country Profile (30) both document a gradual decline in national smoking prevalence following the implementation of tobacco tax reforms, smoke-free legislation, and intensified health education campaigns. Moreover, the Chinese Center for Disease Control and Prevention (CCDC) and National Health Commission have reported substantial improvements in public awareness of smoking hazards and smoking cessation rates since the rollout of the Healthy China 2030 strategy. Incorporating these China-specific findings further underscores that national-level policy interventions have already begun to alter behavioral and epidemiological patterns, aligning with the trends identified in our Joinpoint and BAPC model analyses.

### 4.2 Interpretation of long-term trends

Building on the long-term temporal patterns identified in Sections 3.3 and 3.4, the newly updated BAPC projections further contextualize the observed decline in ASDR since 2010. These projections align with national policy developments particularly the 2015 tobacco tax adjustment and the implementation of smoke-free regulations under the Healthy China 2030 framework which are documented to have strengthened cessation behavior and reduced smoking initiation rates [(32); China CDC, 2022]. Incorporating these epidemiological and policy developments provides a more coherent interpretation of the declining period effects observed in the APC model and allows clearer linkage between statistical findings and their public health implication

This study systematically analyzed the evolving trend of the ischemic stroke burden attributable to smoking in China by integrating Joinpoint and Bayesian Age-Period-Cohort (BAPC) models, providing quantitative predictions of the future burden. In comparison to similar studies worldwide, the unique contribution of this study lies in its use of the GBD (Global Burden of Disease) database to highlight gender differences among middle-aged and elderly men and the projected upward trend in the burden over the next 15 years. This offers policymakers a forward-looking perspective, enabling more precise and targeted tobacco control strategies. Moreover, the study highlights the heightened risks faced by men due to ineffective policy implementation or a resurgence in smoking behavior. Unlike studies that rely solely on historical data, this study integrates past trends with future projections using the BAPC model, addressing a key research gap concerning the future trajectory of smoking-related stroke burden in China. This innovative approach offers a valuable reference for public health interventions in low- and middle-income countries, particularly concerning men's smoking behavior. A growing body of evidence suggests that the gender differences observed in smoking-attributable ischemic stroke mortality in China are driven by multiple interconnected behavioral, cultural, and biological mechanisms. First, the persistently high smoking prevalence among men is strongly shaped by sociocultural norms that associate smoking with masculinity, social bonding, and workplace networking, leading to earlier smoking initiation and heavier lifetime exposure. In contrast, social norms discourage smoking among women, resulting in significantly lower cumulative exposure and delayed uptake. Second, men demonstrate higher rates of hypertension, excessive alcohol consumption, and occupational stress-risk factors that synergistically amplify the cerebrovascular harm caused by smoking. Third, biological differences may further widen the gap; studies indicate that men experience greater smoking-induced endothelial dysfunction and atherosclerotic progression, whereas women may benefit from partial vascular protection before menopause due to estrogen-related mechanisms. Together, these behavioral, cultural, and biological pathways help explain why men show a higher absolute mortality risk and a slower rate of decline despite overall improvements in tobacco control.

### 4.3 Policy implications and targeted interventions

The gender-specific disparities observed in our APC analyses are consistent with prior epidemiological evidence indicating substantially higher cumulative smoking exposure among middle-aged and older Chinese men, driven by entrenched sociocultural norms and sustained high prevalence (National Health Commission, 2021). Furthermore, male populations demonstrate higher rates of hypertension, alcohol use, and occupational stress, which may synergistically amplify smoking-related cerebrovascular risk. These factors, together with slower adoption of cessation interventions among men, likely contribute to the more modest decline in male ASDRs and the projected stabilization rather than continued decline after 2030. Integrating these contextual elements enhances the interpretability of the sex-specific forecast trajectories.

According to this study's predictions, the mortality rate of smoking-attributable ischemic stroke in the male population is expected to rise over the next 15 years, with the increasing smoking rate among women also posing a potential risk. While net drift analysis indicated that the stroke risk for the overall population declined by 1.14% annually in the past, future risk patterns may still be influenced by factors such as aging and persistent smoking behavior within specific birth cohorts. These predictions underscore the need for smoking cessation services to be integrated into the basic public health system, with personalized intervention strategies tailored to different genders and age groups (33). Studies have demonstrated that the burden of smoking-related ischemic stroke has decreased in economically developed regions due to strict tobacco control policies, whereas the burden remains substantial in underdeveloped regions (34). Moreover, as China's economy develops and its population ages, socioeconomic changes have likely influenced smoking behavior and the stroke burden, further impacting the effectiveness of tobacco control policies. Therefore, future tobacco control policies should be optimized to account for the characteristics of different age groups, genders, and regions. By raising tobacco taxes and allocating these funds to public health initiatives, particularly for smoking cessation services targeting high-risk male groups, tobacco consumption can be effectively reduced, and the transformation of the tobacco industry can be facilitated. Furthermore, tobacco control campaigns should be conducted through social media, traditional media, and medical institutions to enhance health education for men and raise awareness of the harms of smoking, particularly among the elderly. Personalized smoking cessation services should be systematically incorporated into health examinations for high-risk middle-aged and elderly groups, with increased availability of smoking cessation medications. Finally, underdeveloped regions should strengthen tobacco control legislation, restrict tobacco advertising, and promote smoking bans in public places to reduce the burden of smoking-related stroke (35–37).

### 4.4 Comparison with international evidence

It is noteworthy that the findings of this study differ somewhat from similar studies conducted in other countries and regions. In comparison to many high-income countries (such as the United States, the United Kingdom, and Australia), China's tobacco control policies still exhibit gaps in enforcement and coverage. In these countries, smoking rates and the associated disease burden have been significantly reduced through measures such as strict tobacco taxes, advertising bans, and comprehensive smoking bans in public places (32, 38). For instance, the United States has seen significant success through the Family Smoking Prevention and Tobacco Control Act, while Australia has notably reduced smoking rates via plain packaging regulations and increased tobacco tax policies (39, 40). By contrast, the smoking-related stroke burden in China continues to rise, particularly among men over the age of 65. Although the Advertising Law of the People's Republic of China has restricted tobacco advertising and promoted a ban on smoking in public places, law enforcement and public health awareness still require strengthening (41). Meanwhile, compared to other low- and middle-income countries (such as India, Indonesia, and Vietnam), China's tobacco control policies have made some progress in urban areas. However, in underdeveloped regions, policy implementation remains inadequate, and significant regional disparities in tobacco control measures persist, similar to the situation in other low- and middle-income countries (42, 43). This study further confirmed these disparities and highlighted the key challenges for future tobacco control efforts. Utilizing the latest GBD 2021 data in conjunction with the BAPC model to project future trends, this study offers a unique perspective on the future prevention and control of ischemic stroke in China, particularly regarding the effective control of male smoking behavior and the reduction of the stroke burden, which holds significant public health importance.

Although previous studies have examined stroke burden or smoking-attributable mortality using GBD data, our study contributes several novel insights. First, we integrate three complementary analytical approaches—Joinpoint regression, APC decomposition, and Bayesian APC forecasting-within a single framework to provide a comprehensive assessment of long-term trends and future projections. Second, we present sex-specific APC effects and cohort-risk patterns not previously described in detail in the Chinese context, offering new evidence on how historical smoking behaviors and demographic transitions shape current mortality trends. Third, by quantifying projected mortality trajectories up to 2035, our study generates forward-looking evidence that allows policymakers to anticipate emerging high-risk groups, particularly older male cohorts.

The policy implications of our findings extend beyond general recommendations for tobacco control. Based on the sex-specific and cohort-specific risk patterns identified, policymakers could prioritize: (1) targeted cessation programs for middle-aged and older male smokers who exhibit persistently elevated cohort risks; (2) strengthened enforcement of smoke-free policies in rural and underserved regions where progress has lagged; and (3) integration of smoking cessation counseling into hypertension and cardiovascular risk management pathways to address synergistic risk factors. These concrete strategies translate our projections into actionable steps that align with China's long-term public health goals and resource constraints.

### 4.5 Limitations

This study is subject to the following limitations. First, the regional data in the GBD database may be incomplete or biased, particularly regarding health statistics from underdeveloped areas of China (44). Second, the age-period-cohort (APC) and Bayesian age-period-cohort (BAPC) models assume linear changes in age, period, and cohort effects, potentially overlooking more complex dynamic changes (45). As a result, this may affect the accuracy of the results to some extent. Nonetheless, this study offers valuable quantitative predictions and policy recommendations regarding the burden of smoking-related ischemic stroke in China by integrating the GBD database with advanced statistical models. These findings serve as an essential foundation for formulating more targeted tobacco control strategies in the future. Future studies should aim to improve data coverage, particularly in underdeveloped areas, and optimize the model to better capture dynamic factors and enhance the accuracy and policy relevance of the predictions. Although the composition of tobacco products may have changed over time, including potential shifts in carcinogen or toxin content, the GBD comparative risk assessment framework accounts for such variations in estimating risk exposure. Nonetheless, future research is warranted to assess the direct impact of evolving tobacco product content on stroke risk in different populations.

Due to limitations in the GBD data structure, we were unable to directly compare trends in smoking-attributable and non-smoking-attributable ischemic stroke mortality within the same dataset. However, existing studies suggest that although overall stroke mortality has been declining, the proportion of deaths attributable to smoking has also decreased, especially among males, potentially as a result of strengthened tobacco control efforts. We have acknowledged this data limitation in our study and recommend that future research should explore this comparison more explicitly to provide a more comprehensive understanding of stroke mortality trends across smoking status groups.

Furthermore, potential regional biases and data incompleteness within the GBD 2021 database may have influenced the precision of our findings and projections. In particular, underreporting or incomplete mortality data from underdeveloped and rural areas of China could lead to an underestimation of smoking-attributable ischemic stroke mortality, especially among older male populations, who exhibit both higher smoking prevalence and limited access to healthcare services. Such data gaps may distort the true magnitude of gender disparities observed in this study. Moreover, regional differences in diagnostic capacity and reporting quality may cause disproportionate representation of urban data, potentially inflating apparent declines in age-standardized death rates (ASDRs) due to better surveillance systems. These biases could also affect the Bayesian Age–Period–Cohort (BAPC) projections, resulting in underestimated future risks in rural male populations. Therefore, while the overall trends and model projections provide valuable insights, they should be interpreted with caution, acknowledging that improvements in data completeness and surveillance represent critical priorities for enhancing future predictive accuracy.

To address the limitation of relying solely on aggregated GBD data without access to individual-level information, we adopted several mitigation strategies. First, we used multiple complementary modeling approaches (Joinpoint, APC, and BAPC) to cross-validate temporal patterns and ensure consistency across methods. Second, we interpreted the results conservatively, avoiding causal inferences and focusing on long-term patterns rather than short-term fluctuations. Third, we emphasized policy implications at the population level, aligning with the ecological nature of GBD data. Although these mitigation steps cannot fully eliminate the constraints of aggregated data, they enhance methodological rigor and the robustness of our inferences.

### 4.6 Conclusion

This study provides a comprehensive assessment of smoking-attributable ischemic stroke mortality in China from 1990 to 2021, highlighting substantial sex-specific differences in both historical trends and future projections. Age-standardized mortality declined steadily over the past decade, with a markedly greater reduction among women, reflecting both low smoking prevalence and favorable shifts in demographic and behavioral determinants. In contrast, male mortality demonstrated only a modest decline, consistent with persistently high smoking prevalence and slower adoption of cessation interventions.

The Bayesian age-period-cohort model suggests that, under current policy conditions, male mortality is expected to stabilize rather than markedly rebound in the coming 15 years, whereas female mortality will remain relatively low. These forecasts indicate that while progress has been made, the burden among men remains substantial and vulnerable to stagnation without strengthened control measures.

Taken together, the findings underscore the critical need for sustained and more targeted tobacco control strategies particularly for middle-aged and older men along with continued improvements in stroke prevention and management. Strengthening enforcement of existing regulations, expanding access to effective cessation support, and reducing regional disparities in tobacco control implementation will be essential to further lowering the burden of smoking-attributable ischemic stroke in China. Continued monitoring and policy evaluation will be required to ensure long-term and equitable reductions in stroke mortality nationwide.

## Data Availability

The datasets presented in this study can be found in online repositories. The names of the repository/repositories and accession number(s) can be found in the article/supplementary material.

## References

[1] Asakawa T Zong L Wang L Xia Y Namba H. Unmet challenges for rehabilitation after stroke in China. Lancet. (2017) 390:121–2. doi: 10.1016/S0140-6736(17)31584-2 28699584

[2] Chen Y Wright N Guo Y Turnbull I Kartsonaki C Yang L . Mortality and recurrent vascular events after first incident stroke: a 9-year community-based study of 0.5 million Chinese adults. Lancet Glob Health. (2020) 8:e580–90. doi: 10.1016/S2214-109X(20)30069-3 32199124 PMC7090905

[3] Peters SAE Huxley RR Woodward M. Smoking as a risk factor for stroke in women compared with men: a systematic review and meta-analysis of 81 cohorts, including 3,980,359 individuals and 42,401 strokes. Stroke. (2013) 44:2821–8. doi: 10.1161/STROKEAHA.113.002342 23970792

[4] Bøttcher M Falk E. Pathology of the coronary arteries in smokers and non-smokers. Eur J Cardiovasc Risk. (1999) 6:299–302. doi: 10.1177/204748739900600504 10534131

[5] GBD 2019 Diseases and Injuries Collaborators. Global burden of 369 diseases and injuries in 204 countries and territories, 1990–2019: a systematic analysis for the Global Burden of Disease Study 2019. Lancet. (2020) 396:1204–22. doi: 10.1016/S0140-6736(20)30925-9 33069326 PMC7567026

[6] Chan KH Xiao D Zhou M Peto R Chen Z. Tobacco control in China. Lancet Public Health. (2023) 8:e1006–e15. doi: 10.1016/S2468-2667(23)00242-6 38000880

[7] Gan Y Wu J Li L Zhang S Yang T Tan S . Association of smoking with risk of stroke in middle-aged and older Chinese. Medicine. (2018) 97:e13260. doi: 10.1097/MD.0000000000013260 30461631 PMC6392934

[8] The Lancet. GBD 2015: from big data to meaningful change. Lancet. (2016) 388:1447–850, e11. doi: 10.1016/S0140-6736(16)31790-1 27733277

[9] Hou S Pang M Zhang Y Xia Y Wang Y Wang G. Assessing tobacco-related ischemic stroke in Pakistan (1990–2019): insights from the Global Burden of Disease Study. Tob Induc Dis. (2024) 22:55. doi: 10.18332/tid/185566 38550907 PMC10973799

[10] Wang M Miao H. Disease burden and related risk factors of esophageal cancer in China and globally from 1990 to 2021, with forecast to 2035: an analysis and comparison. Tob Induc Dis. (2024) 1:22. doi: 10.18332/tid/191389 39091891 PMC11292605

[11] Platzbecker K Voss A Reinold J Elbrecht A Biewener W Prieto-Alhambra D . Validation of algorithms to identify acute myocardial infarction, stroke, and cardiovascular death in German health insurance data. Clin Epidemiol. (2022) 14:1351–61. doi: 10.2147/CLEP.S380314 36387925 PMC9661914

[12] Ren F Shi Z Shen X Xiao G Zhang C. The global, regional, and national burden of stomach cancer attributed to smoking in 204 countries, 1990-2019: a systematic analysis for the Global Burden of Disease Study 2019. Tob Induc Dis. (2024) 1:22. doi: 10.18332/tid/183803 38434517 PMC10907929

[13] Rea F Pagan E Monzio Compagnoni M Cantarutti A Pugni P Bagnardi V . Joinpoint regression analysis with time-on-study as time-scale. Application to three Italian population-based cohort studies. Epidemiol Biostat Public Health. (2022). doi: 10.2427/12616

[14] Zahnd WE James AS Jenkins WD Izadi SR Fogleman AJ Steward DE . Rural–urban differences in cancer incidence and trends in the United States. Cancer Epidemiol Biomarkers Prev. (2018) 27:1265–74. doi: 10.1158/1055-9965.EPI-17-0430 28751476 PMC5787045

[15] Polednak AP. Trends in mortality from COPD in selected U.S. states differing in tobacco control efforts. COPD. (2010) 7:63–9. doi: 10.3109/15412550903499514 20214465

[16] Nistal-Nuño B. Joinpoint regression analysis to evaluate traffic public health policies by national temporal trends from 2000 to 2015. Int J Inj Contr Safety Promot. (2017) 25:128–33. doi: 10.1080/17457300.2017.1341937 28675063

[17] Chernyavskiy P Little MP Rosenberg PS. Correlated poisson models for age-period-cohort analysis. Stat Med. (2017) 37:405–24. doi: 10.1002/sim.7519 28980325 PMC5768446

[18] Murphy CC Yang YC. Use of age-period-cohort analysis in cancer epidemiology research. Curr Epidemiol Rep. (2018) 5:418–31. doi: 10.1007/s40471-018-0174-8 31011507 PMC6474378

[19] Dutey-Magni P. Bayesian sample size determination for planning hierarchical bayes small area estimates. arXiv [Preprint] *arXiv.1802.09388* (2018). doi: 10.48550/arXiv.1802.09388

[20] Gustafson P. Sample size implications when biases are modelled rather than ignored. J Royal Stat Soc Series Stat Soc. (2006) 169:865–81. doi: 10.1111/j.1467-985X.2006.00436.x

[21] Lutz W Sanderson W Scherbov S. The end of world population growth. Nature. (2001) 412:543–5. doi: 10.1038/35087589 11484054

[22] Schmid VJ Held L. Bayesian age-period-cohort modeling and prediction – BAMP. J Stat Softw. (2007) 21:1–15. doi: 10.18637/jss.v021.i08

[23] Feigin VL Norrving B Mensah GA. Global burden of stroke. Circ Res. (2017) 120:439–48. doi: 10.1161/CIRCRESAHA.116.308413 28154096

[24] R Core Team. R: A language and environment for statistical computing. Computing (2011) 1:12–21.

[25] National Cancer Institute. Joinpoint Regression Program, Version 4.9.0.0. Statistical Methodology and Applications Branch, Surveillance Research Program (2021). Available online at: https://surveillance.cancer.gov/joinpoint/

[26] Rue H Martino S Chopin N. Approximate Bayesian inference for latent Gaussian models by using integrated nested Laplace approximations. J Royal Stat Soc Series B. (2009) 71:319–92. doi: 10.1111/j.1467-9868.2008.00700.x

[27] Blangiardo M Cameletti M Baio G Rue H. Spatial and spatio-temporal models with R-INLA. Spat Spatiotemporal Epidemiol. (2013) 4:33–49. doi: 10.1016/j.sste.2012.12.001 23481252

[28] GBD 2019 Risk Factors Collaborators. Global burden of 87 risk factors in 204 countries and territories, 1990-2019: a systematic analysis for the Global Burden of Disease Study 2019. Lancet. (2020) 396:1223–49. doi: 10.1016/S0140-6736(20)30752-2 33069327 PMC7566194

[29] Wickham H François R Henry L Müller K. dplyr: a grammar of data manipulation. R package version 1.0.9. Available online at: https://CRAN.R-project.org/package=dplyr (Accessed October 10, 2025).

[30] World Health Organization (WHO). Report on the Global Tobacco Epidemic 2023: China Country Profile. Geneva: World Health Organization (2023).

[31] Chinese Center for Disease Control and Prevention (China CDC). China Adult Tobacco Survey Report 2022. Beijing: People's Medical Publishing House (2023).

[32] World Health Organization (WHO). WHO report on the global tobacco epidemic 2019: Offer help to quit tobacco use. Geneva: WHO (2019). Available Available online at: https://www.who.int (Accessed September 5, 2025).

[33] Dieleman LA van Peet PG Vos HMM. Gender differences within the barriers to smoking cessation and the preferences for interventions in primary care a qualitative study using focus groups in The Hague, The Netherlands. BMJ Open. (2021) 11:e042623. doi: 10.1136/bmjopen-2020-042623 33514579 PMC7849885

[34] Norrving B Kissela B. The global burden of stroke and need for a continuum of care. Neurology. (2013) 80:S5–12. doi: 10.1212/WNL.0b013e3182762397 23319486 PMC12443346

[35] Crosbie E Sosa P Glantz SA. The importance of continued engagement during the implementation phase of tobacco control policies in a middle-income country: the case of Costa Rica. Tob Control. (2016) 26:60–8. doi: 10.1136/tobaccocontrol-2015-052701 26856614 PMC4977207

[36] Fitzpatrick I Byrne D Gilmore AB Hasan F Cranwell J. Quantifying and characterising tobacco content in the most in-demand streamed series in 10 low/middle-income countries in 2019. Tob Control. (2022) 33:45–51. doi: 10.1136/tobaccocontrol-2022-057278 35750485 PMC10803995

[37] Centers for Disease Control and Prevention (CDC). Tobacco Control Act. U.S. Department of Health & Human Services. Available online at: https://www.cdc.gov/tobacco (Accessed December 17, 2025).

[38] U.S. Food and Drug Administration (FDA). Family Smoking Prevention and Tobacco Control Act. U.S. Department of Health & Human Services. Available online at: https://www.fda.gov/tobacco-products (Accessed December 17, 2025).

[39] Australian Government Department of Health. Tobacco plain packaging. Australian Government. Available online at: https://www.health.gov.au (Accessed December 17, 2025).

[40] Wang Y Ouyang Y Liu J Zhu M Zhao G Bao W . Tobacco use and risk of acute stroke in 32 countries: a cross-sectional analysis of the PURE study. Lancet Glob Health. (2021) 9:e1397–406.

[41] Campaign for Tobacco-Free Kids. China Legal Summary | Tobacco Control Laws (2022). Available online at: https://www.tobaccocontrollaws.org/legislation/country/china/summary (Accessed September 1, 2025).

[42] World Health Organization (WHO) Indonesia. Country Profile: Indonesia Tobacco Control. Available online at: https://www.who.int/indonesia (Accessed September 1, 2025).

[43] Institute for Health Metrics and Evaluation (IHME). GBD Results Tool. Global Health Data Exchange. Available online at: https://ghdx.healthdata.org/gbd-results-tool (Accessed September 1, 2025).

[44] Bell A Jones K. The impossibility of separating age, period and cohort effects. Soc Sci Med. (2013) 93:163–75. doi: 10.1016/j.socscimed.2013.04.029 23701919

[45] Wiśniowski A Smith PWF Bijak J Raymer J Forster JJ. Bayesian population forecasting: extending the lee-carter method. Demography. (2015) 52:1035–59. doi: 10.1007/s13524-015-0389-y 25962866

